# Early Postoperative Simultaneous Occlusion of the Internal and External Iliac Artery Limbs after GORE Excluder Iliac Branch Endoprosthesis Implantation

**DOI:** 10.3400/avd.cr.26-00078

**Published:** 2026-08-29

**Authors:** Akito Kuwano, Hisashi Sato, Akito Setoguchi, Junji Yunoki

**Affiliations:** 1 Department of Cardiovascular Surgery, Kouseikai Hospital, Nagasaki, Nagasaki, Japan; 2 Department of Cardiology, Kouseikai Hospital, Nagasaki, Nagasaki, Japan; 3 Department of Thoracic and Cardiovascular Surgery, Faculty of Medicine, Saga University, Saga, Saga, Japan

**Keywords:** endovascular aneurysm repair, GORE Excluder endoprosthesis, iliac limb occlusion

## Abstract

An 85-year-old man underwent endovascular repair using the Excluder iliac branch endoprosthesis (IBE) for an abdominal aortic aneurysm with a left internal iliac artery aneurysm. He was discharged uneventfully but returned on postoperative day 16 with acute left limb occlusion. Imaging revealed complete occlusion of both the internal and external iliac limbs of the IBE, requiring emergent thrombectomy. Intraoperative assessment demonstrated mechanical compression of the external iliac limb by the internal iliac limb, causing stenosis and thrombosis. This rare complication highlights the importance of meticulous preoperative planning to prevent limb-related events, particularly in patients treated outside the device’s indications.

## Introduction

In the surgical management of abdominal aortic and iliac artery aneurysms, preservation of at least 1 internal iliac artery is recommended to reduce the risk of complications, including spinal cord ischemia, bowel ischemia, sexual dysfunction, and buttock claudication.^1)^ The Excluder IBE (GORE EXCLUDER Iliac Branch Endoprosthesis; W. L. Gore & Associates, Flagstaff, AZ, USA) is a device designed to preserve the internal iliac artery during endovascular aneurysm repair (EVAR). Favorable limb patency has been well documented, and the device is now widely adopted in clinical practice.^2)^ We report an extremely rare case of Excluder IBE in which both the internal and external iliac artery limbs (EILs) occluded in the early postoperative period, and we discuss the potential mechanisms underlying this complication and key considerations for IBE deployment.

## Case Report

**Fig. 1:**
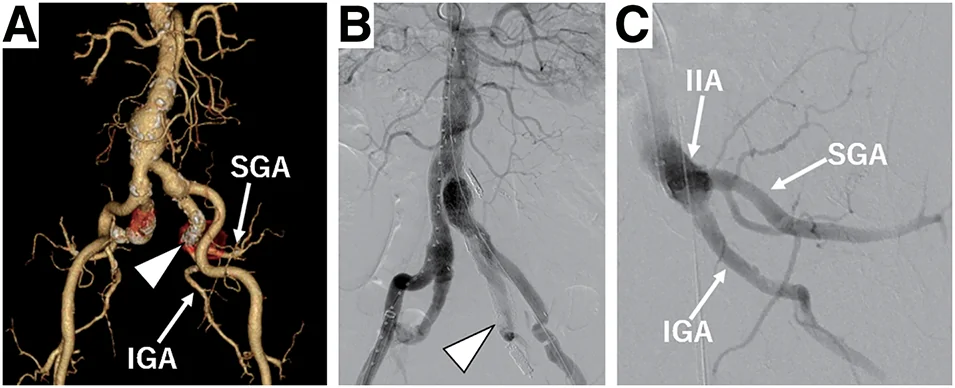
EVAR using the excluder Iliac branch endoprosthesis. EVAR using the Excluder iliac branch endoprosthesis for an abdominal aortic aneurysm and a left internal iliac artery aneurysm (arrowhead) (**A**). Intraoperative angiography reveals no apparent endoleak; however, delayed filling of the left superior gluteal artery is observed (**B**). Intraoperative angiography of the left internal iliac artery demonstrates multiple relatively large branches arising from the superior gluteal artery in close proximity to the internal iliac artery aneurysm (**C**). IGA, inferior gluteal artery; IIA, internal iliac artery; SGA, superior gluteal; EVAR, endovascular aneurysm repair

**Fig. 2:**
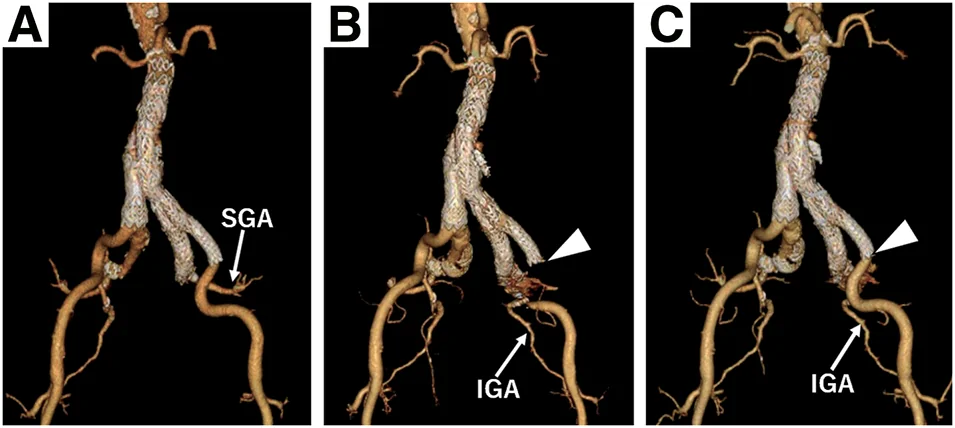
CT findings. Contrast-enhanced CT performed on postoperative day 7 demonstrates patency of both the internal and external iliac limbs (**A**). CT performed on postoperative day 16 reveals complete occlusion of both the internal and external iliac limbs of the iliac branch endoprosthesis (arrowhead). Distal perfusion of the external iliac artery is preserved via collateral circulation, and the inferior gluteal artery is opacified through collateral pathways (**B**). CT performed after reintervention demonstrates restored patency of the external iliac limb (arrowhead), whereas the internal iliac limb remains occluded. The inferior gluteal artery continues to be opacified via collateral circulation (**C**). IGA, inferior gluteal artery; SGA, superior gluteal artery; CT, computed tomography

An 85-year-old man with no significant past medical history underwent EVAR using the Excluder IBE to treat an abdominal aortic aneurysm (maximum diameter, 36 mm) and a left internal iliac artery aneurysm (maximum diameter, 37 mm) while preserving perfusion to the left internal iliac artery (IIA) (**Fig. 1A**). As part of the IIA preservation procedure, the inferior gluteal artery was embolized, and the internal iliac artery limb (IIL) component, which had a distal diameter of 12 mm, was extended using a VBX 8L (GORE VIABAHN VBX Balloon Expandable Endoprosthesis; W. L. Gore & Associates) with a distal landing in the superior gluteal artery, which measured approximately 5 mm in diameter. The EIL component of the IBE, which had a distal diameter of 12 mm, was also deployed. Intraoperative angiography revealed no apparent endoleak; however, delayed filling of the left superior gluteal artery was observed (**Fig. 1B**). The postoperative ankle brachial index (ABI) was 1.24 on the right and 1.23 on the left. Contrast-enhanced computed tomography (CT) performed on postoperative day 7 demonstrated no evidence of an endoleak, and both the IIL and EIL remained patent (**Fig. 2A**). The postoperative course was uneventful, and the patient was discharged on postoperative day 11.

**Fig. 3:**
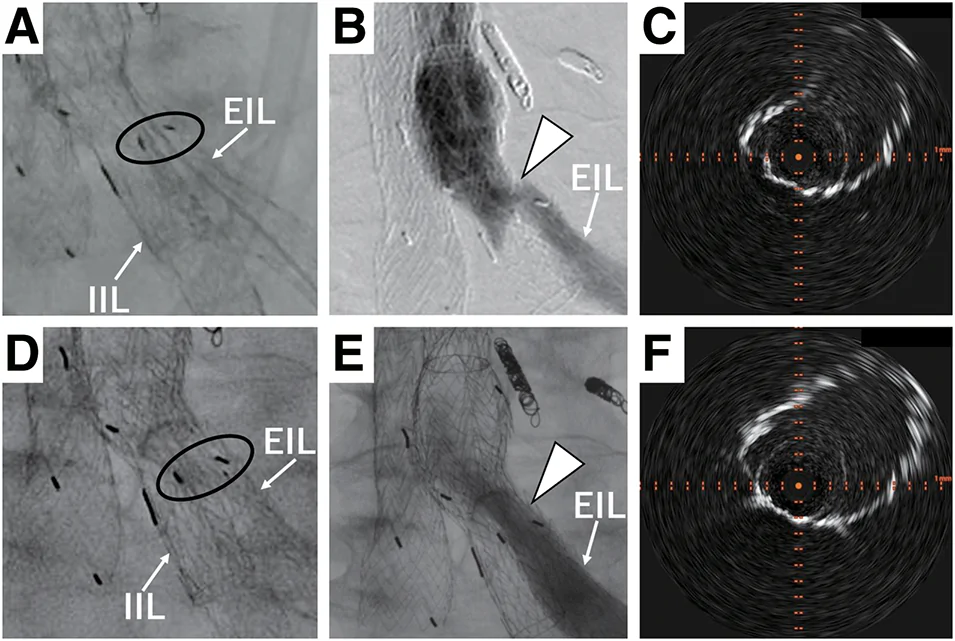
Angiographic and IVUS findings at reintervention. Angiography at reintervention demonstrates stenosis at the orifice of the external iliac limb caused by compression from the internal iliac limb (**A**, circled area; **B** arrowhead). IVUS reveals flattening and narrowing of the orifice (**C**). After stent deployment, angiography demonstrates resolution of the stenosis (**D**, circled area) and restoration of blood flow (**E**, arrowhead). IVUS confirms adequate stent expansion (**F**). EIL, external iliac limb; IIL, internal iliac limb; IVUS, intravascular ultrasound

He presented emergently to our hospital 5 days after discharge with weakness and numbness in the left lower limb. Physical examination revealed that the left lower limb was cold with mild cyanosis, and the left femoral, popliteal, dorsalis pedis, and posterior tibial pulses were not palpable. The ABI was 1.21 on the right, whereas it was undetectable on the left. Contrast-enhanced CT revealed complete occlusion extending from the left limb of the Excluder to both the IIL and EIL of the IBE, while distal flow in the external iliac artery (EIA) was maintained via collateral circulation (**Fig. 2B**). Through a left groin incision, emergent thrombectomy was performed using a 4-Fr Fogarty catheter inserted via the femoral artery, yielding a large amount of fresh, dark-red thrombus. Follow-up angiography after thrombectomy demonstrated stenosis at the orifice of the EIL, which was dilated with a 10-mm balloon (**Fig. 3A** and **3B**). Although the stenosis improved, intravascular ultrasound (IVUS) revealed that the orifice remained flattened and narrowed (**Fig. 3C**). No stenosis or dissection attributable to the distal end of the stent graft limb in the left EIA was observed. Given the risk of recurrent limb-related events, a 12-mm bare-metal stent (SMART 12 × 40 mm; Cordis, Miami Lakes, FL, USA) was deployed, and adequate stent expansion and restoration of blood flow were confirmed by angiography and IVUS (**Fig. 3D**–**3F**).

Following the reintervention, dual antiplatelet therapy with aspirin and clopidogrel was initiated to reduce the risk of recurrent stent occlusion. The ABI was 1.21 on the right and 1.20 on the left. Contrast-enhanced CT demonstrated patent flow in the EIL despite persistent occlusion of the IIL, with the inferior gluteal artery opacified via collateral circulation (**Fig. 2C**). At 5 months after the reintervention, he remained free from buttock claudication and recurrent limb-related events.

## Discussion

The Excluder IBE facilitates preservation of the IIA during EVAR for abdominal aortic and iliac artery aneurysms. In a multicenter study conducted by Schneider et al., the reported 5-year limb patency rates were 100% for the external iliac limb and 95.1% for the internal iliac limb, demonstrating excellent long-term durability.^2)^ Conversely, the IBE has specific anatomic requirements, as specified in the instructions for use (IFU), and it has been reported that only approximately 30% of Japanese patients strictly meet these criteria.^3)^ Yunoki et al. reported favorable limb patency rates in a cohort that included patients treated outside the IFU, among whom only 30% satisfied all IFU criteria.^4)^ These findings have contributed to the broader adoption of the IBE in contemporary practice. Furthermore, in recent years, the utility of iliac branch graft devices for the treatment of internal iliac artery aneurysms (IIAAs) has also been demonstrated.^5)^ The present case was outside the IFU owing to a narrow common iliac artery measuring 36 mm in length, with a minimum diameter of 14 mm and a maximum diameter of 19.7 mm at the EIA–IIA bifurcation, as well as a concomitant IIAA and a limited distance between the lowest renal artery and the iliac bifurcation. Although the anatomy was outside the IFU, the use of the IBE was considered a reasonable option because complete embolization of the major branches of the left IIA was considered technically challenging (**Fig. 1C**) and preservation of perfusion to the superior gluteal artery was desirable. Consequently, the IIAA was successfully managed by embolization of the inferior gluteal artery and extension of the IIL component into the superior gluteal artery using a VBX 8L; however, postoperative simultaneous occlusion of both the internal and external iliac limbs occurred. Because the contralateral internal iliac artery was preserved, an alternative strategy consisting of embolization of the major branches of the left IIA followed by extension of the stent graft into the EIA could also have been considered.

Several studies have reported adverse events involving the IIL following IBE implantation, including stenosis and occlusion. Notably, most such events occur within 30 days postoperatively and are generally attributable to anatomic and technical factors rather than to device durability.^6,7)^ Several anatomic and procedural factors have been implicated in limb-related events, including a small IIA diameter, excessive oversizing of the IIL component, landing zone tortuosity or atherosclerotic disease, compromised outflow, compression of the IIL component secondary to stenosis of the IIA, and the absence of antiplatelet therapy.^6)^ In addition, DeRoo et al. identified an IIA diameter of ≤10 mm as a significant risk factor for limb-related events.^7)^ In the present case, the superior gluteal artery was relatively small (approximately 5 mm in diameter), and the use of a VBX 8L may have resulted in relative oversizing, leading to sluggish flow on intraoperative angiography and predisposing the IIL to early postoperative occlusion. Furthermore, because antiplatelet therapy is not routinely administered after EVAR at our institution, none was initiated after the index EVAR in the present case; however, its use in patients with risk factors for limb-related events may help mitigate the risk of subsequent complications.

Reports of adverse events involving the EIL after IBE implantation are scarce. We found no previous reports describing early simultaneous occlusion of both the IIL and EIL after Excluder IBE implantation. In a series of 81 patients encompassing 105 treated iliac arteries, Méndez Fernández et al. reported 2 cases of the EIL occlusion, which were attributed to marked vascular tortuosity.^8)^ Schneider et al. described acute occlusion of the EIL on postoperative day 1 that required thrombectomy, although the precise etiology remained unclear.^9)^ van Sterkenburg et al. documented asymptomatic stenosis of the EIL treated with balloon-expandable stent placement in the late postoperative period; however, detailed clinical information was limited.^10)^ In the present off-IFU case, deployment of the iliac branch component within a narrow common iliac artery with a minimum diameter of 14 mm likely resulted in mechanical compression of the EIL by the IIL, leading to stenosis at the EIL orifice and subsequent early postoperative occlusion. Notably, this lesion was not apparent on postoperative CT but was clearly delineated by IVUS. The ABI remained within the normal range in the early postoperative period, suggesting that the degree of stenosis was initially mild. However, after occlusion of the IIL occurred through the aforementioned mechanism, reduced flow in the EIA likely resulted in sluggish hemodynamics and subsequent thrombosis. These findings suggest that even mild EIL stenosis not detectable on CT may contribute to limb-related events; therefore, additional assessment with complementary imaging modalities, including intraoperative CT and IVUS, may be useful. In selected cases, adjunctive bare-metal stent placement within the EIL component may help preserve limb patency. Taken together, we believe that the indication for the Excluder IBE use should be carefully considered in off-IFU cases, particularly those involving a narrow common iliac artery, underscoring the need for meticulous preoperative planning and careful postoperative follow-up.

## Conclusions

We present a case in which Excluder IBE implantation was complicated by early postoperative occlusion of both the internal and EILs. Particularly in patients outside the IFU with a narrow common iliac artery, meticulous preoperative planning that considers the morphological features of the IBE is crucial to minimize the risk of limb-related events. Furthermore, in patients with risk factors for limb-related events, careful assessment using complementary imaging modalities, adjunctive stent placement, and tailored antiplatelet therapy may help optimize limb patency, and careful postoperative follow-up is warranted.

## References

[1] Ogino H, Iida O, Akutsu K, et al. JCS/JSCVS/JATS/JSVS 2020 guideline on diagnosis and treatment of aortic aneurysm and aortic dissection. Circ J 2023; 87: 1410–621. 37661428 10.1253/circj.CJ-22-0794

[2] Schneider DB, Matsumura JS, Lee JT, et al. Five-year outcomes from a prospective, multicenter study of endovascular repair of iliac artery aneurysms using an iliac branch device. J Vasc Surg 2023; 77: 122–8. 35842202 10.1016/j.jvs.2022.07.006

[3] Sugimoto M, Takahashi N, Niimi K, et al. Anatomical suitability of the GORE EXCLUDER iliac branch endoprosthesis in Japanese patients with common iliac aneurysms treated by standard EXCLUDER endografts. Ann Vasc Surg 2018; 50: 179–85. 29499354 10.1016/j.avsg.2017.11.071

[4] Yunoki J, Kamohara K, Koga S, et al. Early results of expanding the anatomical indications for using a Gore iliac branch endoprosthesis to treat aortoiliac and iliac aneurysms. Surg Today 2021; 51: 1028–35. 33237376 10.1007/s00595-020-02183-4

[5] Noel-Lamy M, Jaskolka J, Lindsay TF, et al. Internal iliac aneurysm repair outcomes using a modification of the iliac branch graft. Eur J Vasc Endovasc Surg 2015; 50: 474–9. 26188719 10.1016/j.ejvs.2015.05.021

[6] van der Veen D, Holewijn S, Bellosta R, et al. One year outcomes of an international multicentre prospective cohort study on the Gore Excluder iliac branch endoprosthesis for aorto-iliac aneurysms. Eur J Vasc Endovasc Surg 2021; 62: 177–85. 34144884 10.1016/j.ejvs.2021.04.006

[7] DeRoo E, Harris D, Olson S, et al. Conformability of the GORE EXCLUDER iliac branch endoprosthesis is associated with freedom from adverse iliac events. J Vasc Surg 2021; 74: 1558–64.e1. 34082005 10.1016/j.jvs.2021.05.026

[8] Méndez Fernández A, Fernández Noya J, Mosquera Arochena NJ, et al. Results of the Galician registry in the treatment of complex aortoiliac aneurysms with GORE^®^ EXCLUDER^®^ Iliac Branch Endoprosthesis (GALIBER). Vascular 2022; 30: 620–7. 34114523 10.1177/17085381211025173

[9] Schneider DB, Milner R, Heyligers JMM, et al. Outcomes of the GORE iliac branch endoprosthesis in clinical trial and real-world registry settings. J Vasc Surg 2019; 69: 367–77.e1. 30064841 10.1016/j.jvs.2018.05.200

[10] van Sterkenburg SM, Heyligers JM, van Bladel M, et al. Experience with the GORE EXCLUDER iliac branch endoprosthesis for common iliac artery aneurysms. J Vasc Surg 2016; 63: 1451–7. 27230243 10.1016/j.jvs.2016.01.021

